# Elevated Expression of the Long Noncoding RNA IFNG-AS1 in the Peripheral Blood from Patients with Rheumatoid Arthritis

**DOI:** 10.1155/2020/6401978

**Published:** 2020-01-30

**Authors:** Huiyong Peng, Shuting Ren, Yingzhao Liu, Huimin Zhou, Xinyi Tang, Jun Yang, Jie Tian, Ping Xu, Huaxi Xu, Shengjun Wang

**Affiliations:** ^1^Department of Laboratory Medicine, The Affiliated People's Hospital, Jiangsu University, Zhenjiang 212002, China; ^2^Department of Laboratory Medicine, The PLA 904 Hospital, Wuxi 214044, China; ^3^Department of Endocrinology, The Affiliated People's Hospital, Jiangsu University, Zhenjiang 212002, China; ^4^Institute of Laboratory Medicine, Jiangsu Key Laboratory of Laboratory Medicine, Jiangsu University School of Medicine, Zhenjiang 212013, China; ^5^Department of Laboratory Medicine, The Fifth People's Hospital of Suzhou, Suzhou, China

## Abstract

Long noncoding RNAs (lncRNAs) have been increasingly recognized as key immune molecules that participate in the pathogenesis of autoimmune diseases. Previous studies have demonstrated that the lncRNA Ifng-AS1, a key scaffold that contributes to the transcription of IFN-*γ*, depends on T-bet for active transcription in Th1 cells. However, the effect of its human ortholog, IFNG-AS1, on the pathogenesis of rheumatoid arthritis (RA) remains unclear. In this study, we found that the transcript level of lncRNA IFNG-AS1 was increased in the peripheral blood of RA patients. IFNG, as a target gene of IFNG-AS1, was overexpressed and positively correlated with the transcript level of IFNG-AS1 in the RA patients. Our data also showed that the transcript level of T-bet was upregulated and positively correlated with IFNG-AS1 expression. T-bet regulated the transcription of IFNG-AS1 in human CD4^+^ T cells in vitro. Furthermore, strong positive correlations were observed between the increased transcript level of IFNG-AS1 and the serum level of rheumatoid factor, the erythrocyte sedimentation rate, and the C-reactive protein in RA patients, and patients positive for anticyclic citrullinated peptide antibodies had increased levels of IFNG-AS1. Finally, receiver operating characteristic (ROC) curve analysis suggested that IFNG-AS1 might be a potential biomarker of RA. Taken together, our findings indicated that IFNG-AS1, guided by T-bet, is augmented in the peripheral blood of RA patients and may play a critical role in the pathogenesis of RA by regulating the expression of IFNG.

## 1. Introduction

Rheumatoid arthritis (RA) is an autoimmune disease characterized by chronic relapsing-remitting inflammation of the peripheral synovial joints, resulting in the progressive destruction of the joint cartilage and erosion of bone tissue and ultimately leading to physical disability [1]. The serological features of RA patients include elevated production of rheumatoid factor (RF), an increased erythrocyte sedimentation rate (ESR), and increased levels of C-reactive protein (CRP). Some patients also test positive for anticyclic citrullinated peptide antibodies (anti-CCP Ab) [2, 3]. This complex and expanding disease is believed to be caused by genetic predisposition, environmental factors, and immunopathogenesis [4]. Although T cells and B cells are both involved in the disease process, CD4^+^ T helper cells and their cytokines are now believed to play a critical role in the induction and propagation of inflammatory conditions [5, 6].

Interferon *γ* (IFN-*γ*) is a soluble protein that belongs to one subset of the interferon family, and its transcript is composed of four exons. The gene encoding this protein is located at chromosome 12 (IFNG) or chromosome 10 (Ifng). This pleiotropic protein is a homodimer that binds to the IFN-*γ* receptor (IFN-*γ*R) to mediate downstream signaling events [7]. IFN-*γ* is mainly secreted by cells of both the innate and adaptive immune systems, including T cells and natural killer (NK) cells, and, to a lesser extent, by other cell types, such as macrophages, dendritic cells (DCs), and B cells [8]. This molecule is the main inflammatory cytokine that distinguishes the Th1 lineage from other CD4^+^ T subsets [9]. To control infection, CD8^+^ T cells secrete IFN-*γ*, and it is produced by CD4^+^ T helper 1 (Th1) cells that play an essential role in several immune processes, including the clearance of intracellular invasive pathogens, inflammation, and autoimmune diseases [10–12]. Naive CD4^+^ T cells do not produce IFN-*γ*, but they differentiate into effector Th1 cells, which can produce IFN-*γ* in the presence of antigenic stimulation. This process depends on the activation of JAK/STAT pathway components, including the transcription factors STAT1 and STAT4 in the presence of interleukin 12 [13, 14]. T-bet is the key transcription factor that promotes the transcription of Ifng as well as silencing of the gene encoding interleukin 4 (IL-4) in Th1 cells [15, 16]. Accumulated evidence has demonstrated that IFN-*γ* produced by Th1 cells is involved in the pathogenesis of RA [17–19]. However, the underlying mechanism of elevated IFN-*γ* in RA patients is still poorly understood.

Long noncoding RNAs (lncRNAs) have recently been shown to be key regulators of gene expression. These molecules have low evolutionary sequence conservation and are highly prevalent in the eukaryotic transcriptome [20]. Although the majority of these molecules have been identified in the mammalian genome by bioinformatics analyses of transcriptomic data, only a few of their functions have been characterized [21, 22]. In recent years, lncRNAs have been widely reported to play critical roles in the immune system [23–25]. The lncRNA transcript Ifng-AS1, formally known as Tmevpg1 or NeST, was initially identified by Vigneau et al. [26]. This lncRNA is expressed in CD4^+^ T cells, CD8^+^ T cells, and NK cells, and its human ortholog is located at the opposite strand of the IFN-*γ*-encoding gene (IFNG). Recent studies have demonstrated that Tmevpg1 is a key regulator in the expression of Ifng by Th1 cells [27]. However, the role of IFNG-AS1 in the pathogenesis of RA is unknown.

In this study, we aimed to identify whether IFNG-AS1 is dysregulated in RA patients. We found that IFNG-AS1 was strongly increased and positively correlated with the level of IFNG, as well as the disease severity, in RA patients. IFNG-AS1 was transcriptionally regulated by T-bet in human CD4^+^ T cells. These findings provide novel insight into the role of IFNG-AS1 in the pathogenesis of RA.

## 2. Materials and Methods

### 2.1. Subjects and Samples

Thirty-one rheumatoid arthritis patients, aged forty-two to seventy-four years, including twenty-three females and eight males, were enrolled in the study. All RA patients were diagnosed by clinical manifestations and auxiliary examination and fulfilled the American College of Rheumatology (ACR) 1987 and the European League Against Rheumatism (EULAR) 2009 revised criteria for the classification of RA. Eight patients with osteoarthritis (OA), aged forty-four to seventy-six years, including six females and two males, were enrolled in the present study. Thirty age- and sex-matched healthy subjects were included as controls. All the healthy subjects showed normal serological features, including RF, anti-CCP-Ab, ESR, and CRP, and had no history of joint disease or other autoimmune diseases. The number of peripheral leukocytes was within the normal range. The main clinical characteristics of these patients and the healthy controls are summarized in Table 1. Peripheral blood samples were obtained from all the patients and the healthy controls.

All samples were collected after the subjects provided informed consent in accordance with the regulations and with the approval of the research ethics committee of the Affiliated People's Hospital of Jiangsu University.

Data correspond to the arithmetic mean ± SD. +: positive; -: negative; M: male; F: female.

### 2.2. Cell Isolation and Purification In Vitro

Human peripheral blood mononuclear cells (PBMCs) were isolated by density-gradient centrifugation over Ficoll-Hypaque solution (Haoyang Biological Technology Co., Tianjin, China) and stored at -80°C until use for quantitative real-time PCR (qRT-PCR). Human CD4^+^ T cells were isolated from the PBMCs using human CD4 microbeads (Miltenyi Biotec GmbH, Bergisch Gladbach, Germany) as previously described [28]. Human CD4^+^ T cells were cultured in RPMI-1640 medium (Gibco, California, USA) supplemented with 10% fetal bovine serum (Gibco) for transfection.

### 2.3. RNA Isolation and qRT-PCR

Total RNA was isolated from the PBMCs with TRIzol reagent (Invitrogen, California, USA) according to the manufacturers' instructions. The cDNA was synthesized with random primers and a ReverTra Ace®qPCR RT kit (Toyobo, Osaka, Japan). qRT-PCR was performed in triplicate using Bio-Rad SYBR Green Super Mix (Bio-Rad, Hercules, USA). The primer sequences are shown in Table 2. *β*-Actin was used as a reference gene to quantitatively analyze the genes of interest in the study. Data were analyzed using Bio-Rad CFX Manager software.

### 2.4. Small Interfering RNA Transfection

Small interfering RNA (siRNA) (RiboBio, Guangzhou, China) was designed against the sequence of T-bet. Nonspecific scramble siRNA was used as a negative control (NC). The purified human CD4^+^ T cells were transfected with T-bet siRNA or NC at 50 nM using Entranster-R (Engreen Biosystem, Co., Ltd., Beijing, China) according to the manufacturers' instructions for 24 hours in the presence of 0.5 *μ*g/mL functional anti-human CD3 mAb plus 2 *μ*g/mL functional anti-human CD28 mAb (Miltenyi Biotec GmbH).

### 2.5. Statistical Analysis

Data were analyzed with GraphPad Prism version 5 software (GraphPad Software, Inc., San Diego, USA). The statistical significance of differences between the groups was determined via *t*-tests or ANOVA. Correlations between variables were determined by Pearson's correlation coefficients. ROC curve analysis was performed to evaluate the diagnostic value of IFNG-AS1, which was dysregulated in the PBMCs of the RA patients compared to those of the healthy controls. A *p* value < 0.05 was considered significant (^∗^*p* < 0.05, ^∗∗^*p* < 0.01, and ^∗∗∗^*p* < 0.001).

## 3. Results

### 3.1. Increased Expression of IFNG-AS1 Correlates with the Clinical Disease Severity in the RA Patients

**Figure 1:**
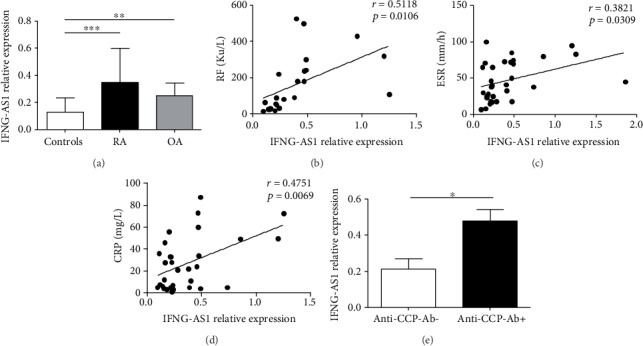
Increased expression of IFNG-AS1 correlates with the clinical disease severity in the RA patients. (a) The transcript levels of IFNG-AS1 in the PBMCs from the RA patients and the healthy controls were detected by qRT-PCR. The correlations between the transcript level of IFNG-AS1 and the concentration of RF (b), the ESR (c), and the CRP level (d) in the RA patients are shown. (e) The relative expression of IFNG-AS1 in the PBMCs from the anti-CCP Ab- and anti-CCP-Ab+ RA patients was determined. Each data point represents an individual subject, and the horizontal lines show the mean. ^∗^*p* < 0.05; ^∗∗^*p* < 0.01; ^∗∗∗^*p* < 0.001.

IFNG-AS1 is comprised of four exons, is located at chromosome 12q15 adjacent to IFNG, and helps facilitate Th1 cell-dependent Ifng expression both in humans and in mice. To determine whether IFNG-AS1 is abnormally expressed in RA patients, we detected the transcript level of IFNG-AS1 via qRT-PCR. We used the OA patients as a disease control in the study. As shown in Figure 1(a), the transcript levels of IFNG-AS1 were significantly increased in the PBMCs from the RA patients compared with those of the healthy controls, and upregulation of IFNG-AS1 expression was also observed in the OA patients. Moreover, positive correlations were shown between the transcript level of IFNG-AS1 and the level of RF (*r* = 0.5118, *p* = 0.0106) (Figure 1(b)), the ESR (*r* = 0.3821, *p* = 0.0309) (Figure 1(c)), and the level of CRP (*r* = 0.4751, *p* = 0.0069) in the RA patients (Figure 1(d)). We also found that IFNG-AS1 was substantially greater in the anti-CCP-Ab-positive patients than in the anti-CCP-Ab-negative patients (Figure 1(e)). These data showed that abnormal IFNG-AS1 expression is associated with the process of RA.

### 3.2. The Transcript Level of IFNG-AS1 Positively Correlates with the Elevated Level of IFNG in the RA Patients

**Figure 2:**
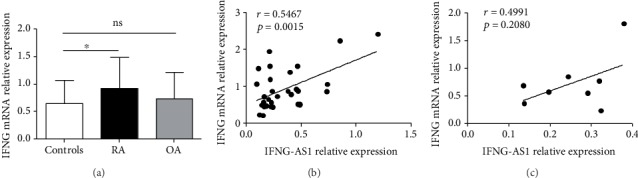
IFNG-AS1 expression positively correlates with the elevated level of IFNG in the RA patients. (a) The IFNG mRNA levels in the PBMCs from the RA patients, the OA patients, and the healthy controls were detected by qRT-PCR. (b) The correlation between the transcript level of IFNG-AS1 and the transcript level of IFNG in the RA patients is shown. (c) The correlation between the transcript level of IFNG-AS1 and the mRNA level of IFNG in the OA patients is shown. Each data point represents an individual subject, and the horizontal lines show the mean. ^∗^*p* < 0.05.

Accumulated evidence has demonstrated that IFN-*γ* produced by Th1 cells is involved in the pathogenesis of RA. IFNG, as a transcript of IFN-*γ*, is essential for the differentiation and function of Th1 cells. Here, we found that the level of IFNG was significantly upregulated in the PBMCs from the RA patients (Figure 2(a)), and a notable positive correlation between the transcript level of IFNG-AS1 and the elevated level of IFNG (*r* = 0.5467, *p* = 0.0015) was shown in the RA patients (Figure 2(b)). The level of IFNG, by contrast, was not changed in the OA patients (Figure 2(a)), and there was no correlation between the level of IFNG and the level of IFNG-AS1, which was also increased in the OA patients (Figure 2(c)). These data suggested that the level of IFNG-AS1 is associated with IFNG expression in RA patients.

### 3.3. Positive Correlations between the Elevated Level of T-Bet and the Level of IFNG-AS1 in the RA Patients

**Figure 3:**
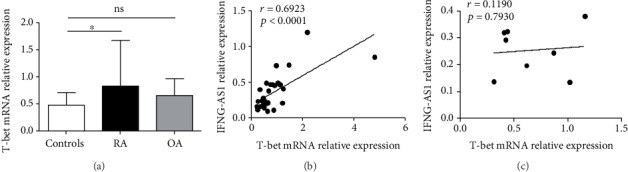
Positive correlations between the elevated levels of T-bet and IFNG-AS1 in the RA patients. (a) The relative mRNA levels of T-bet in the PBMCs from the RA patients, the OA patients, and the healthy controls were detected by qRT-PCR. (b) The correlation between the transcript level of T-bet mRNA and the level of IFNG-AS1 in the RA patients is shown. (c) The correlation between T-bet mRNA expression and IFNG-AS1 expression in the OA patients is shown. Each data point represents an individual subject, and the horizontal lines show the mean. ∗*p* < 0.05.

T-bet is a crucial transcription factor that contributes to the transcription of IFNG in Th1 cells and regulates the transcription of Ifng-as1 in a mouse model [29]. To investigate the relationship between T-bet and IFNG-AS1 in the RA patients, we measured the level of T-bet via qRT-PCR. We found that the level of T-bet was significantly increased in the PBMCs from the RA patients (Figure 3(a)). The level of T-bet positively correlated with the transcript level of IFNG-AS1 (*r* = 0.6923, *p* < 0.0001) (Figure 3(b)). However, no correlation was found between the level of IFNG-AS1 and the level of T-bet in the OA patients (Figure 3(c)), similar to the healthy controls (Figure 3(a)). These data showed that T-bet might participate in the transcription of IFNG-AS1 in the RA patients.

### 3.4. Effect of T-Bet on IFNG-AS1 Transcription in Human CD4^+^ T Cells

**Figure 4:**
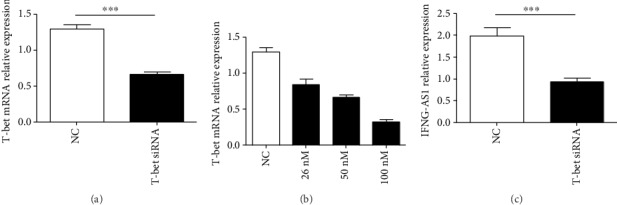
Effect of T-bet on IFNG-AS1 transcription in human CD4^+^ T cells. Human CD4^+^ T cells were purified from the PBMCs by magnetic beads and transfected with the T-bet-specific siRNA and the negative control (50 nM) in the presence of functional anti-human CD3 mAb and anti-human CD28 mAb to detect the transcript levels of T-bet mRNA and IFNG-AS1. (a) The mRNA level of T-bet was detected by qRT-PCR after transfection. (b) The mRNA level of T-bet was detected after transfection with the T-bet-specific siRNA in a dose-dependent manner. (c) The transcript level of IFNG-AS1 was detected by qRT-PCR after transfection. The results are shown as the mean ± SD of three independent experiments, and the horizontal lines show the mean. ^∗∗∗^*p* < 0.001.

To confirm whether T-bet participates in IFNG-AS1 transcription, we isolated human CD4^+^ T cells and transfected them with T-bet-specific siRNA. The T-bet-specific siRNA interfered with the transcription of T-bet in a dose-dependent manner (Figures 4(a) and 4(b)). The level of IFNG-AS1 was obviously reduced in the CD4^+^ T cells transfected with the T-bet-specific siRNA compared with the negative control (Figure 4(c)). Together, these results indicated that T-bet regulated the transcription of IFNG-AS1 in human CD4^+^ T cells.

### 3.5. Potential Diagnostic Value of IFNG-AS1 in RA

**Figure 5:**
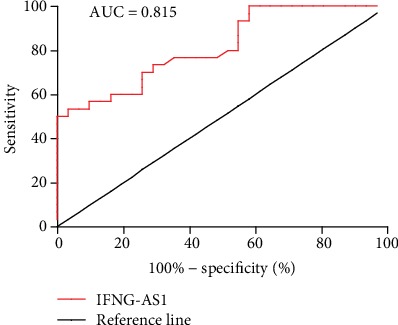
Potential diagnostic value of IFNG-AS1 in RA. ROC curve analysis using IFNG-AS1 to distinguish the RA patients was performed. ROC curve analysis was performed to show the AUC of IFNG-AS1.

ROC curve analysis was performed to evaluate the potential value of IFNG-AS1 in the peripheral blood from the RA patients. The area under the ROC curve (AUC) was up to 0.815 [95% confidence interval (CI) = 0.711-0.919, *p* < 0.001], and the sensitivity and specificity were 53.33% and 96.77%, respectively (Figure 5). These data showed that IFNG-AS1 might be a potential biomarker of RA.

## 4. Discussion

LncRNAs are increasingly appreciated as important regulators involved in various autoimmune diseases via multiple mechanisms to promote or inhibit the transcription of cell-specific genes. Previous studies have demonstrated that Ifng-AS1 is a lncRNA transcript that influences Ifng transcription during Th1 differentiation. This molecule was initially identified as a candidate gene at the Tmevpg3 locus that controls mouse intracranial viral infection following inoculation with Theiler's virus [26]. In contrast to other Th cell subclasses, Tmevpg1 is selectively expressed in Th1 cells [27]. However, the role of IFNG-AS1 in RA patients is unclear. In this study, we found that IFNG-AS1 was highly expressed in RA patients and positively correlated with elevated transcript levels of IFNG, which is an IFN-*γ*-encoding gene. Our previous studies have shown that IFNG-AS1 knockdown results in a considerable reduction in the transcript level of IFNG and the proportion of Th1 cells in vitro [30, 31]. Interestingly, the expression of Ifng-AS1 was undetectable in effector CD8^+^ T cells under Th1 culture conditions, which also produced a significant amount of IFN-*γ* [27]. Our previous data showed that there was no correlation between the transcript level of IFNG-AS1 and the percentage of CD8^+^IFN-*γ*^+^ T cells in the peripheral blood of patients with Hashimoto's thyroiditis. Hence, we speculated that IFNG-AS1 might contribute to the production of IFN-*γ* by Th1 cells in the RA patients. However, the underlying regulatory mechanism by which IFNG-AS1 influences the expression of IFNG in Th1 cells is still unclear. NeST was overexpressed in SJL/J mice and found to recruit WDR5 protein, a component of the histone H3 lysine 4 methyltransferase complex, to the IFN-*γ* locus to catalyze the methylation of histone H3 at lysine 4, a marker of active gene expression in activated CD8^+^ T cells [32]. The discrepancy in Ifng-AS1 expression between the Th1 cells and the CD8^+^ T cells is possibly due to different animal models and culture conditions, as well as different mechanisms of IFN-*γ* production by CD4^+^ T cells and CD8^+^ T cells. Further studies are needed to investigate the underlying mechanisms of IFN-*γ* regulated by IFNG-AS1 in Th1 cells, although NeST was shown to regulate the epigenetic marking of the IFN-*γ*-encoding chromatin in CD8^+^ T cells. OA is a degenerative joint disease characterized by degraded cartilage, moderate synovial inflammation, alteration of the bony structure, pain, and impaired mobility [33]. Although the clinical symptoms of OA and RA patients are similar, their pathogenic mechanisms are diverse. In this study, we found that the transcript level of IFNG-AS1 was also upregulated in the OA patients, but there was no correlation between the transcript levels of IFNG-AS1 and IFNG, which was similar in the OA patients and the healthy volunteers. We speculated that IFNG-AS1 overexpression was not associated with the IFN-*γ*-induced inflammatory response in OA. These results imply that IFNG-AS1 participates in the pathogenesis of RA via catalyzing IFN-*γ* production.

Most studies focus on the downstream regulatory mechanisms of lncRNAs, and few have reported the upstream regulatory mechanisms. A previous study showed that T-bet induces active transcription of Tmevpg1 by guiding epigenetic remodeling of the Tmevpg1 enhancer locus, leading to the recruitment of the transcription factors NF-*κ*B and Ets-1 in mice [29]. Based on these findings, we speculated that the abnormal expression of IFNG-AS1 might be regulated by T-bet in RA patients. As expected, an increased transcript level of T-bet was found in the peripheral blood from the RA patients. Furthermore, a significant positive correlation was found between the level of T-bet and the transcript level of IFNG-AS1 in the RA patients. By contrast, there was no correlation in the OA patients. In vitro, T-bet knockdown by a specific siRNA resulted in a significant reduction of IFNG-AS1 in human CD4^+^ T cells. These data combined with previous studies suggest that T-bet may be involved in the production of IFN-*γ* via regulating IFNG-AS1 expression in RA patients, providing new insight into the regulatory mechanism of T-bet in human Th1 cells.

RA is an autoimmune disease caused by the combined effect of multiple autoantibodies. The elevated serum concentrations of RF and anti-CCP-Ab are the most valuable manifestations of RA, and some patients have a high ESR and high levels of CRP. These clinical parameters could be used to indicate the development and prognosis of RA. IFN-*γ* production by CD4^+^ T cells drives the generation of IgG and IgM, while RF and anti-CCP-Ab belong predominantly to the IgM and IgG classes, respectively [34, 35]. In this study, we analyzed the correlation between the transcript level of IFNG-AS1 and the serum level of clinical diagnostic manifestations. Positive correlations were found between the transcript level of IFNG-AS1 and the levels of RF, ESR, and CRP. Moreover, IFNG-AS1 was significantly upregulated in the anti-CCP-Ab-positive patients. These results suggested that IFNG-AS1 expression, to some extent, mirrored the disease severity of RA.

Extensive clinical data have demonstrated that RF is not specific, while anti-CCP-Ab is not sensitive, for RA diagnosis [36, 37]. In addition, anti-CCP-Ab cannot be used as an indicator to assess the severity of RA patients [38]. It is necessary to identify new biomarkers for RA patients. Hence, we explored the potential diagnostic value of IFNG-AS1 in RA. Our results indicated that the AUC of IFNG-AS1 in the PBMCs of the RA patients was up to 0.815 and the sensitivity and specificity were 96.8% and 53.3%, respectively. These data indicated that IFNG-AS1 in PBMCs might be used as a potential biomarker, which combined with the detection of RF and anti-CCP-Ab could improve the sensitivity and specificity of the diagnosis of RA.

## 5. Conclusions

Our results demonstrated that IFNG-AS1 is significantly augmented in RA patients and regulated by the high expression of T-bet. The significant correlation between overexpressed IFNG-AS1 and IFNG, as well as disease severity, indicates that IFNG-AS1 may play an essential role in the development of RA via regulating the expression of IFNG. The ROC curve analysis showed that IFNG-AS1 is a potential biomarker of RA. Further studies with larger cohorts of RA patients are needed.

## Figures and Tables

**Table 1:** Clinical features of the patients and the healthy controls included in the study.

	RA patients	OA patients	Healthy controls	Range
Number	31	8	30	
Gender (M/F)	8/23	2/6	6/24	
Age (years)	58 ± 16	60 ± 16	50 ± 20	
RF (IU/mL)	150 ± 153	8 ± 4	5 ± 3	0-20
CRP (mg/L)	19.1 ± 22.3	15.9 ± 26.2	0.78 ± 0.76	0-5
ESR (mm/h)	49 ± 31	17 ± 18	3 ± 1	0-20
Anti-CCP-Ab (+/-)	25/6	—	—	

**Table 2:** Primer sequences of the genes in the study.

Primer ID	Nucleotide	Sequence (5′-3′)	
T-bet	NM_013351.2	Forward	TTGAGGTGAACGACGGAGAG
		Reverse	GGCATTCTGGTAGGCAGTCA

IFNG	NM_000619.3	Forward	GAGTGTGGAGACCATCAAGGA
		Reverse	TGTATTGCTTTGCGTTGGAC

IFNG-AS1	NR_104125.1	Forward	GCTGATGATGGTGGTGGCAATCT
		Reverse	TTAGCAGTTGGTGGGCTTCT

*β*-Actin	NM_001101.5	Forward	GAGTGTGGAGACCATCAAGGA
		Reverse	TGTATTGCTTTGCGTTGGAC

## Data Availability

The data used to support the findings of this study are included within the article.
